# Patient-Centered Care in Breast Cancer Genetic Clinics

**DOI:** 10.3390/ijerph15020319

**Published:** 2018-02-12

**Authors:** Anne Brédart, Amélie Anota, Julia Dick, Violetta Kuboth, Olivier Lareyre, Antoine De Pauw, Alejandra Cano, Dominique Stoppa-Lyonnet, Rita Schmutzler, Sylvie Dolbeault, Jean-Luc Kop

**Affiliations:** 1 Supportive Care Department, Psycho-Oncology Unit, Institut Curie, 26 Rue d’Ulm, 75005 Paris CEDEX 05, France; olivier.lareyre@curie.fr (O.L.); sylvie.dolbeault@curie.fr (S.D.); 2 Psychopathology and Health Process Laboratory, University Paris Descartes, EA 4057, 71 Avenue Edouard Vaillant, 92774 Boulogne-Billancourt, France; 3 Quality of Life and Cancer Clinical Research Platform, Methodology and Quality of Life in Oncology Unit (INSERM UMR 1098), CHU Besançon, 25030 Besançon, France; aanota@chu-besancon.fr; 4 Familial Breast and Ovarian Cancer Centre, Cologne University Hospital and Faculty of Medicine, Kerpener Str. 34, 50931 Cologne, Germany; julia.dick@uk-koeln.de (J.D.); violetta.kuboth@web.de (V.K.); rita.schmutzler@uk-koeln.de (R.S.); 5 Cancer Genetic Clinic, Institut Curie, 26 Rue d’Ulm, 75005 Paris CEDEX 05, France; antoine.depauw@curie.fr (A.D.P.); dominique.stoppa-lyonnet@curie.fr (D.S.-L.); 6 Clinical and Health Psychology Department, University Autónoma of Barcelona, Carrer de Ca N’Altayó, s/n, 08193 Barcelona, Spain; alejandra.cano@e-campus.uab.cat; 7 Centre de Recherche en Épidémiologie et Santé des Populations (CESP), University Paris-Sud, UVSQ, INSERM, University Paris-Saclay, 16 Avenue Paul Vaillant-Couturier, 94807 Villejuif CEDEX, France; 8 Department 2LPN-CEMA, Université de Lorraine, 23 boulevard Albert 1er - BP 60446 - 54001 NANCY CEDEX, France; jean-luc.kop@univ-lorraine.fr

**Keywords:** breast cancer, genetic risk, risk counseling, psychosocial needs, distress, service delivery, culture

## Abstract

With advances in breast cancer (BC) gene panel testing, risk counseling has become increasingly complex, potentially leading to unmet psychosocial needs. We assessed psychosocial needs and correlates in women initiating testing for high genetic BC risk in clinics in France and Germany, and compared these results with data from a literature review. Among the 442 counselees consecutively approached, 212 (83%) in France and 180 (97%) in Germany, mostly BC patients (81% and 92%, respectively), returned the ‘Psychosocial Assessment in Hereditary Cancer’ questionnaire. Based on the Breast and Ovarian Analysis of Disease Incidence and Carrier Estimation Algorithm (BOADICEA) BC risk estimation model, the mean BC lifetime risk estimates were 19% and 18% in France and Germany, respectively. In both countries, the most prevalent needs clustered around the “living with cancer” and “children-related issues” domains. In multivariate analyses, a higher number of psychosocial needs were significantly associated with younger age (b = −0.05), higher anxiety (b = 0.78), and having children (b = 1.51), but not with country, educational level, marital status, depression, or loss of a family member due to hereditary cancer. These results are in line with the literature review data. However, this review identified only seven studies that quantitatively addressed psychosocial needs in the BC genetic counseling setting. Current data lack understandings of how cancer risk counseling affects psychosocial needs, and improves patient-centered care in that setting.

## 1. Introduction

Breast cancer (BC) is a major public health concern for women, with almost 1.7 million new BC diagnoses worldwide in 2012 [1]. Among these BC patients, 10% to 20% present with a BC family history, and two decades ago, *BRCA1* and *BRCA2* were identified as major BC susceptibility genes [2]. Women who carry a mutation in the *BRCA1* or *BRCA2* gene face up to a 72% and 44% risk of developing breast or ovarian cancer, respectively, by the age of 80 [3]. When already affected with BC, these women present an increased risk of contralateral BC [3]. 

The identification of a pathogenic variant in highly penetrant genes such as *BRCA1* or *BRCA2* provides options for early detection or prevention. There is evidence that a risk-reducing bilateral mastectomy decreases the risk of BC by 85% to 100% [4], and that risk-reducing bilateral salpingo-oophorectomy reduces the risk of ovarian cancer by 69% to 100%, and the risk of BC by 37% to 100% [4]. When a pathogenic variant is identified, genetic testing may be offered to relatives.

Recently, additional genetic factors have been identified, including rare variants with “moderate” (*CHEK2, ATM, BARD1* and *RAD51D*) to “high” (*PALB2*) BC risk [5]. Thanks to technological advances, instead of testing the *BRCA1* and *BRCA2* genes alone, panels of genes may now be simultaneously tested, at a reduced cost and faster turnaround [6]. As a result, over the past few years, these tests have entered oncologic genetic clinics [7,8,9].

National guidelines recommend the inclusion of cancer genetic testing within a framework of genetic counseling [10]. This healthcare discipline is defined as “the process of helping people understand and adapt to the medical, psychological, and familial implications of genetic contributions to disease” [11]. Among tasks such as cancer risk assessment, informed consent collection, the disclosure of genetic test results, and medical recommendations, genetic counseling comprises psychosocial assessment [12]. Thus, the relevance of a ‘patient-centered care approach’ addressing psychosocial needs in this clinical setting should be underlined.

Psychosocial needs refer to psychological and social difficulties, and the type and extent of help a patient actually needs or expects in order to manage these difficulties [13]. Potential needs in cancer genetics may be motivated by specific issues related to the cancer genetic risk, counseling, and testing [14], and may request additional help from a clinical geneticist, genetic counselor, gynecologist, breast surgeon, or psychosocial worker. In past decades, there has been increased interest in needs assessment in cancer care [15], and in cancer genetics; specific questionnaires have been developed in order to address these concerns and adjust cancer care as necessary [16,17].

Genetic counseling confronts large quantities of information involving statistic and genetic concepts, which are generally imprecise and complex, and are sources of uncertainty [18]. Moreover, genetic counselors tend to communicate unidirectionally, providing large pieces of information, and at the same time often neglecting to enquire about counselees’ needs [19], leaving them with potentially unfulfilled needs after the consultation [20,21].

In the gene panel testing environment, genetic counseling is further complicated, as information must be provided on possible new quantitative results (i.e., “moderate” cancer risk with unwell defined guidance for clinical management [22]), an increased number of variants of uncertain clinical significance (VUS) [23], or the possibility of secondary or incidental results [24]. In addition, the involvement of non-genetic factors in BC risk [25] further complicates counseling.

Assessing psychosocial needs during the care trajectory allows for clarifying what type of interventions (i.e., depending on specific needs) or for which population (i.e., depending on its characteristics) the provision of specific health care resources is necessary [26]. Variations across countries in the BC genetic risk counseling process exist, e.g., in terms of risk communication [27] or clinical recommendation [22]. Patterns of psychosocial needs are expected to vary according to these variations.

Psychosocial needs may also reveal based on counselees’ sociodemographic, family history, and clinical or psychosocial characteristics. If their psychosocial needs are similar across counselees’ characteristics, but are different across countries, this implies that the mode of counseling delivery is associated with needs, and this may have implications for clinical practice according to specific settings [28].

To our knowledge, only two United States (US) studies [29,30] reported on psychosocial outcomes in women at high genetic risk of BC undergoing gene panel testing. Initial (pretest) genetic counseling generally provides information allowing counselees to make informed decisions regarding testing and the receipt of genetic information. The present study addressed the psychosocial needs of women at a high genetic risk of BC undergoing genetic counseling in French and German cancer genetic clinics in order to highlight possible unmet care needs before actual genetic panel testing.

Specifically, we aimed to:

(1) Assess the prevalence of moderate and high psychosocial needs in women undergoing genetic counseling and testing for BC risk in a French and a German cancer genetic clinic, and compare them between the two countries;
(2) Explore sociodemographic, clinical, psychological, and geographical (i.e., the country setting) factors associated with the number of moderate and high psychosocial needs in these women; and
(3) Compare these results with empirical data extracted from a literature review addressing the domains and specific items of psychosocial needs that are most frequently reported along the cancer genetic counseling and testing process.

## 2. Materials and Methods

This study protocol was approved in France by the Comité consultatif sur le traitement de l’information en matière de recherche dans le domaine de la santé (CCTIRS: Consultative committee for information management in health research—No. 16.314, France), and by the Commission Nationale Informatique et Libertés (CNIL: French Information Technology and Privacy Commission), and in Germany by the Ethics Committee of the University Hospital of Cologne. All of the recruited women provided written informed consent.

### 2.1. Participants and Procedure

From October 2016 to July 2017, women over the age of 18 years, who were either healthy or affected with a primary non-metastatic breast cancer, and eligible for BC gene panel testing, were consecutively recruited at the cancer genetic units of the Institut Curie (Paris, France) and the University Hospital of Cologne (Cologne, Germany). A minimum sample size of 200 was required to allow for comparisons between the two country cancer genetic services. Women with a recurrent BC, a personal history of ovarian cancer, or a major psychiatric disorder were not included.

The study objectives were explained to the women on the day of the initial cancer genetic counseling visit, and when they agreed to participate, they were given questionnaires to fill in at home, either in paper or online, and to return within the next two weeks. Questionnaires not completed or received within 28 days after the genetics consultation were considered missing. If necessary, 14 days after the consultation, one reminder was made by telephone call.

### 2.2. Questionnaire and Data Collected 

#### 2.2.1. Psychosocial Outcome

The ‘Psychosocial Aspects of Hereditary Cancer’ (PAHC) questionnaire is comprised of 26 items organized into six problem domains, i.e., needs related to hereditary predisposition, practical issues, family and social issues, living with cancer, emotions, and children-related issues [17]. Participants are asked to rate each question on a scale from “not at all” (1), “a little” (2), “quite a bit” (3), or “a lot” (4). A “not applicable” option is provided to some items (e.g., items regarding children-related issues). The PAHC items were translated and adapted into French and German according to international guidelines [31], and were pilot tested in the respective country’s cancer genetic clinic. The internal consistency of the overall PAHC 26-item scale was 0.87.

#### 2.2.2. Independent Variables

General distress (i.e., anxiety and depression) was measured by the Hospital Anxiety and Depression Scale. This scale comprises anxiety (HADS-Anxiety) and depression (HADS-Depression) subscales, and has French [32] and German [33] versions. 

Women were also asked if they had lost family member(s) due to breast or ovarian cancer.

To compute the objective risk estimates of breast and of ovarian cancer up to the age of 80 years, the Breast and Ovarian Analysis of Disease Incidence and Carrier Estimation Algorithm (BOADICEA) web application (BWA v3, University of Cambridge, Cambridge, UK) [34] was used. 

Additional data were collected on the sociodemographic characteristics provided by the patient, and the clinical data obtained from the medical record.

### 2.3. Statistical Analysis

Baseline sociodemographic and clinical characteristics of the participants were described using the mean (standard deviation) or median (range) for continuous variables, and the number (percentage) for qualitative variables. We used the Student *t*-test for quantitative data and the Chi-square test for categorical data to compare study participants among the French and the German respondents, and between the age of overall respondents and non-respondents. Response frequencies were computed for each PAHC item. We computed the frequency (percent) of individuals reporting moderate to high needs (at least “quite a bit” responses) per item. A Chi-square test was performed to compare the percentage of patients with a moderate to high need per item between the two countries.

Multivariate regression analyses were performed on the number of needs (rated at least “quite a bit”) per individual, as a dependent variable. Psychosocial needs were taken as a whole, considering that the PAHC structural validity was not yet confirmed in these PAHC language versions (work in process). The country sample, age, educational level (secondary school or below versus superior education), marital status (married or partnered versus single, separated, divorced or widowed), parental status (having children or not), HADS-anxiety and HADS-depression scores (continuous variables), health status (affected or not with a present or past BC diagnosis), and family history (having lost a blood family member from breast or ovarian cancer) were assessed as potential predictors in univariate analyses. The collinearity between variables was tested in univariate analysis; variables presenting collinearity were not simultaneously included in the same multivariate model. A stepwise selection approach was then used to select the final multivariate model.

### 2.4. Literature Review Procedures

A systematic search of the PubMed/Medline database was conducted. We selected papers published between January 2000 (before that time, *BRCA1/2* psychosocial outcomes were mainly addressed on researched cohorts rather than in “real” clinical practice [35]) to 17 October 2017 (last check), assessing the overall psychosocial difficulties or needs of women who had a high breast or ovarian cancer genetic risk, and considering only observational quantitative studies, using genetics-specific, multidimensional measures (keyword combinations, see Supplementary Materials Table S1).

All of the titles and abstracts were inspected for relevance, excluding papers such as protocol descriptions, literature reviews, studies addressing the period of referral or following cancer risk management decision-making, men only, healthcare professionals, genetic testing for breast or ovarian treatment decision-making, or controlled (randomized) trials. Thirteen papers were rejected on full text as they only addressed a specific outcome (e.g., general or specific distress, quality of care perception) (11 papers) or generic difficulties/needs measures such as the SF-36 (two papers). The two remaining papers [36,37] were analyzed in addition to five studies identified from a manual search [30,38,39,40,41], for a total of seven inspected studies. 

Two authors (Anne Brédart and Jean-Luc Kop) extracted the study descriptive data (study country, sample size, response rate, study population age and gender, timing of assessment, type of cancer genetic risk, cancer diagnosis status, and psychosocial need measure; see Supplementary Materials Table S2), and the prevalence of needs (the number of patients that expressed individual need items), and identified significant relationships between the various factors and psychosocial needs within each study. Additionally, the psychosocial needs measured were described based on the original publication (which measured the aim and targeted population, number of items, scales/domains, developmental and psychometric information, and scoring method; see Supplementary Materials Table S3).

## 3. Results

### 3.1. Samples’ Characteristics

Table 1 displays the characteristics of the French and German samples. Among the 442 counselees approached, 212 (83%) in France and 180 (97%) in Germany provided evaluable PAHC responses. The French and German respondents had a mean age (standard deviation) of 47.8 (12.0) and 47.7 (10.2) years, and 171 (81%) and 165 (92%) were affected with BC, respectively. These women were either under treatment or in remission. The mean (standard deviation) levels of anxiety and depression were 7.94 (3.82%) and 3.81 (3.03%) and, 7.52 (4.07%) and 4.61 (3.96%) respectively, in the French and German samples. Based on the BOADICEA BC risk estimation model [34], the mean (standard deviation) percent of BC lifetime risk estimates by age 80 was 19.2 (11.6%) and 18.0 (9.1%) in France and Germany, respectively.

Overall respondents’ and non-respondents’ mean (standard deviation) age was 47.8 (11.2) and 45.5 (11.9), respectively (the difference was not statistically significant). Compared to the German respondents, the French respondents were significantly less often affected with BC (*p* = 0.001), and presented a higher level of education (*p* < 0.0001) and a lower depression level (*p* = 0.03).

### 3.2. Prevalence of Moderate to High Psychosocial Needs

Out of the over 26 potential psychosocial needs assessed with the PAHC, seven needs were reported by more than 50% of the French women, and five needs were reported by more than 50% of the German women (% prevalence of women rating the presence of each need at least “quite a bit”) (Table 2). Among the 10 most prevalent needs, five clustered around the “living with cancer” domain in the French and German samples, two around “children-related issues”, two around “family and social issues”, and one around “hereditary predisposition” in the French sample; and two around “emotion”, two around “hereditary predisposition”, and one around “children-related issues” in the German sample. Nine needs were reported as significantly more severe by women from France than Germany, whereas two needs were reported as significantly more severe by women from Germany than France. The mean (standard deviation) number of needs were 7.5 (5.3%) and 6.3 (5.3%), respectively, in the French and German samples, which was significantly different (*p* < 0.01).

### 3.3. Correlates of Psychosocial Needs

In multivariate analyses, a significantly higher number of needs was observed in younger women (b = −0.05; *p* = 0.01), as well as in those with a higher level of anxiety (b = 0.78; *p* < 0.0001) and those with children (b = 1.51; *p* = 0.002) (Table 3). A trend towards a higher number of needs was also observed in women affected with BC (b = 1.05; *p* = 0.08). The sample country, marital status, education level, depression level, and loss of family members due to breast or ovarian cancer showed no statistically significant association. The variance in the number of needs explained by the regression model was 40%.

### 3.4. Description of Studies from the Literature Review

The seven studies available were from different Western countries (Australia [36], Austria [37], Netherlands [40], Norway [39], the United Kingdom (UK) [38], and the US [30,41]) (Supplementary Materials S2). With one exception [38], studies were cross-sectional. Five studies addressed breast or ovarian cancer genetic risk [30,36,37,39,41], and the two others reported on various hereditary syndromes [38,40]. Five studies comprised samples with 50% or more counselees affected with cancer [30,37,39,40,41]. Four studies [30,37,39,41] used the ‘Multidimensional Impact of Cancer Risk Assessment’ (MICRA) to assess counselees’ psychosocial difficulties [16], and the three others specifically used the ‘Genetic Risk Assessment Coping Evaluation’ (GRACE) [38,42], the ‘Psychosocial Assessment in Hereditary Cancer’ (PAHC) [17,40], or a modified version of a psychosocial needs questionnaire for BC survivors [36]. Among these questionnaires, only the MICRA provided information on scale validity (structure) and reliability (internal consistency) [16] (Supplementary Materials S3).

### 3.5. Prevalence of Needs from the Literature Review

Table 4 describes the psychosocial needs, as expressed at the initial cancer genetic consultation one month later, or in the medium or long-term after the genetic test result disclosure, depending on the study. At the initial consultation and in counselees from various hereditary cancer syndromes, using the PAHC [17], the most prevalent needs were clustering around “living with cancer” [40], and using the GRACE [42], these were related to family, hereditary predisposition, or practical issues, over two assessment times one month apart [38].

After the disclosure of the *BRC*A1/2 test result, using the MICRA questionnaire [16], BjØrnslett et al. [39] reported a higher level and a different hierarchy of needs prevalence in *BRCA*1/2 carriers compared to non-carriers (e.g., “frustrated that there are no definite cancer prevention guidelines for me”; 41%/ninth highest prevalent need, versus 38%/third highest prevalent need). Around two years after the BRCA1/2 test disclosure, Farrelly et al. [36] found that 50% of women or more presented needs in the three issues: “fear of developing cancer”, “family communication”, and “information on cancer risk management options”. Finally, at around seven years, after the BRCA1/2 test disclosure, using the MICRA scales [16], uncertainty appeared to be the most prevalent need, as expressed by 59% of *BRCA*1/2 tested women, whatever the *BRCA*1/2 test result [41].

### 3.6. Correlates of Needs from the Literature Review

A significant increase in needs was found in younger counselees [36], women with children [40], carriers of a deleterious mutation [30,37,39,41], and those who had received a VUS test result [30]; further correlates were a shorter time since the initial counseling [38] or genetic test disclosure [36,41], distress [39,40], previous contact with a psychosocial worker [40], and lower social support [36]. 

A number of tested factors, including demographics [39,41], clinical characteristics [36,37,39,40,41], family history [36,39], and assessment timing [39], were not significantly associated with needs.

Aspects of the cancer genetic counseling delivery (e.g., psychosocial assessment, risk communication, or counseling) in relation to psychosocial needs were not investigated in the selected reviewed studies (Table 5).

## 4. Discussion

This study primarily aimed to assess the prevalence of psychosocial needs in women initiating genetic counseling for high BC risk in a French and a German cancer genetic clinic, and explore correlates of increased needs, including the country setting. We compared these results to data extracted from a literature review in order to shed light on implications for research and clinical applications.

A substantial proportion of women in both countries perceived significant difficulties in the “living with cancer” domain, which referred to living with both actual cancer as well as the threat of personal or familial cancer diagnoses. In addition, they also experienced concerns in the “family and social issues” domain, representing the interpersonal aspects of the cancer genetic risk. Interventions should thus focus on these particular difficulties in both institutions. Besides, independently from the country, the overall number of needs was found to be higher in younger women, in those having children, or those presenting increased anxiety; therefore, attention should be given to the needs of these women in particular.

Specific differences were also observed between the two country samples. For instance, compared to the German sample, a higher proportion in the French sample worried about children-related issues. For example, women attending the French cancer genetic clinic more frequently expressed “guilt about the chance of passing on genetic alterations to their children” than the German sample; this may suggest that women in the French genetic clinic may need additional counseling for dealing with this feeling of guilt. In contrast, women attending the German cancer genetic clinic more frequently expressed preoccupations related to the hereditary predisposition, such as “worries about the choice of possible preventive options (screening versus surgery)”, which points to the need for additional support in decision-making with regard to management options in the German clinic.

Significant differences (i.e., education level, BC status) between the two country samples may require specific clinical implications by clinic setting. As only the actual BC diagnosis status appeared to be (to some extent) related to a higher number of needs, interventions should predominantly emphasize the needs of women affected with BC.

Our results are in line with the information obtained from the literature review, confirming a high prevalence of psychosocial needs following genetic counseling, including aspects in relation to the cancer diagnosis [40] or family and social issues [38] after the initial cancer genetic consultation. 

Other reviewed studies investigated the prevalence of these needs over time, showing that these needs may remain high (above 50%), independent of the genetic test result: 59% of women worried about the “implications of being at increased risk of cancer for other family members” one month after the initial genetic consultation [38]; 84% of *BRCA*1/2 carriers and 69% of non-carriers “worried about their risk of getting cancer/again” [39]; 57% of *BRCA*1/2 carriers “dealt with fears about developing cancer” at about two years after the genetic test result disclosure [36]; and 59% felt confronted with “uncertainty” about seven years after receiving the genetic test result [41]. This underlines the importance of monitoring psychosocial needs over a longer period of time in individuals confronted with BC genetic risk.

The literature review data found that younger age [36], having children [40], and anxiety [39,40] correlated with psychological needs, thus supporting our findings. To gain further insights into the psychosocial needs of women along the process of BC gene panel testing, we are currently planning a follow-up assessment on psychosocial needs after the women’s receipt of the genetic test result. In line with the literature, we expect significant differences between women according to the genetic test result received (positive, negative, or VUS) in terms of intensity [30,37,41] or hierarchy [39] of needs.

This report comprises a number of limitations. Our study samples mainly comprised BC patients; therefore, the results may not reflect the psychosocial needs of healthy women that are at a high BC genetic risk (e.g., female relatives) undergoing genetic counseling. Moreover, we assessed a limited number of needs correlates, thus potentially missing out on other relevant needs. Further, so far, our data is cross-sectional, i.e., no causal conclusions can be drawn. Hence, in order to provide more specific clinical recommendations, further research should address additional factors in longitudinal study designs. The impact of recent advances in BC risk genetic testing and counseling requires the monitoring of counselees’ well-being. Future research should address the effects of different genetic counseling delivery models, such as the risk communication style on counselees’ psychosocial needs.

Only two quantitative studies that addressed overall psychosocial needs were identified from the literature review based on the chosen databases and keywords. In fact, the psychometric assessment of needs happens to be a relatively new area of research in the cancer field [15]. In the genetic counseling context, this outcome has hardly been considered [43,44,45]. The development of a standard questionnaire to assess psychosocial needs in the cancer genetics population has only recently been implemented [17]. 

Among the seven studies selected, the population samples varied (i.e., in terms of hereditary cancer syndromes, cancer diagnosis, gender, assessment timing), and so, beyond general observations such as high needs prevalence regarding the cancer experience or in relation to younger age and anxiety, making comparisons and drawing conclusions on more specific observations between studies remain problematic. 

Moreover, four different needs questionnaires were employed. Most of them assessed the severity of difficulties, concerns or worries [38,40], and positive or negative emotions [39,41]. Only Farrelly et al. [36] used a questionnaire that directly assessed the presence and intensity of needs, whereas only such an instrument can pinpoint the patient’s or counselee’s expectations with regard to counseling. Furthermore, the data obtained from these different questionnaires were reported in different ways (i.e., either item by item, or by domain or scale), and only one questionnaire (MICRA [16]) evidenced psychometric properties.

## 5. Conclusions

A high prevalence of psychosocial needs clustering around the personal and familial experience of cancer, and the interpersonal aspects of hereditary cancer, were observed among women undergoing BC genetic counseling in a cancer genetic clinic in France and Germany. An increased number of needs were observed in younger, more anxious women, and in those having children, independently from the country setting. This suggests that these younger women in particular may need additional counseling during the course of BC genetic testing. Specific needs were also observed in each country clinic, underlying the relevance of needs assessment in the clinical care setting. A literature review supported these findings, and also pointed at future research directions with regards to BC genetic counseling practices. We should move forward to provide standardized and valid instruments that allowing unambiguous scoring and reporting of counselees’ psychosocial needs within and across cancer genetic clinics. Moreover, in line with recent advances in cancer genetics and risk counseling, future research should focus on the genetic service delivery examining different models of risk communication and counseling that may influence psychosocial needs. Health care specialists, like physicians, nurses, counselors, and psychologists who attend to women at high genetic risk of breast cancer should be trained to identify and respond to their psychosocial needs. In this way the benefits expected from new cancer genetics discoveries and technologies may also manifest in clinical genetics settings by optimally responding to counselees’ needs and ensure a patient-centered care in that setting.

## Figures and Tables

**Table 1:** Sociodemographic and clinical characteristics of the two samples.

	French Respondents (N = 212)	German Respondents (N = 180)	*p*-Value
Age (years)			
Mean (SD)	47.8 (12.0)	47.7 (10.2)	NS
Median (range)	48 (21–78)	48.5 (23–74)	
Education level (%)			
Secondary school or below	66 (31.1)	127 (70.6)	<0.0001
Superior education	144 (67.9)	51 (28.3)	
Missing data	2 (0.94)	2 (1.11)	
Marital status (%)			
Married/partnered	148 (69.8)	120 (66.7)	NS
Others (widowed, separated/divorced, single/never married)	63 (29.7)	59 (32.8)	
Missing data	1 (0.47)	1 (0.56)	
Having children (%) (Yes)	168 (79.3)	128 (71.1)	0.06
Missing data			
Personal breast cancer (%) (Yes)	171 (80.7)	165 (91.7)	0.001
Missing data	0 (0)	1 (0.01)	
Breast cancer lifetime risk			
BOADICEA estimates			
Mean (SD)	19.2 (11.6)	18.0 (9.1)	NS
Median (range)	17.5 (0.8–82.9)	16.5 (1.5–81.1)	
HADS-Anxiety			
Mean (SD)	7.94 (3.82)	7.52 (4.07)	NS
Median (range)	8 (1–19)	7 (0–19)	
Missing data	9	2	
HADS-Depression			
Mean (SD)	3.81 (3.03)	4.61 (3.96)	0.03
Median (range)	3 (0–14)	3 (0–20)	
Missing data	6	3	

NS: not statistically significant; BOADICEA Breast and Ovarian Analysis of Disease Incidence and Carrier Estimation Algorithm; HADS: Hospital Anxiety and Depression Scale; SD: standard deviation.

**Table 2:** Prevalence of participant’s responses to the Psychosocial Aspects of Hereditary Cancer (PAHC) items (% rated at least quite a bit) in the French and German study samples.

Prevalence of Needs	TOTAL (N = 392)	French Sample (N = 212)	German Sample (N = 180)
PAHC items ^$^ % *rated at least quite a bit*		%	%
Worried about the chance of being a carrier of a genetic mutation (HP)	41	40	43
Worried about having to choose whether or not to go for genetic counseling and testing (HP)	6	4	8
Worried about the choice of possible preventive options (screening or surgery) (HP) *	27	22	33
Worried about coping with the (future) DNA test results (HP)	26	24	29
Worried about (fulfilling) your plans for having children (HP) **	11	16	6
Worried about the impact of genetic testing on your daily life (PI)	20	21	18
Worried about the impact of genetic testing on obtaining insurance or mortgage (PI) ****	18	27	9
Misunderstood by partner/family/social circle with respect to genetic testing (FSI)	3	3	3
Bothered by lack of support about genetic testing from partner, family, or your social circle (FSI)	3	4	2
Worried about immediate family’s functioning because of genetic testing (FSI) *	12	8	16
Worried about the contact with family members about genetic testing (FSI) **	6	10	3
Worried about coping with cancer within the family (FSI) ****	43	61	25
Burdened by feelings of responsibility towards family members related to genetic testing (FSI) ****	30	40	20
Anxious (E) *	22	26	18
Tense (E)	24	21	28
Depressed (E)	11	8	13
Insecure about the future (E)	31	27	35
Concerned about life and death (E)	33	31	35
Emotionally burdened that family members have cancer (LWC) ****	54	71	36
Emotionally burdened by losing a family member because of cancer (LWC)	80	83	77
Emotionally burdened by the diagnosis or treatment for cancer (LWC)	56	52	61
Worried about the chance of getting cancer (again) (LWC)	64	66	62
Worried about the chance that family members will get cancer (LWC)	69	72	65
Guilty about the chance of passing possible genetic alterations onto children (CRI) ****	30	39	20
Worried about telling children the results (CRI)	27	28	26
Worried about the chance of children developing cancer (CRI) *	72	79	65
Number of needs **			
Mean (SD) Median (Min–Max)	7.84 (5.00) 7 (0–24)	7.54 (5.31) 7 (0–24)	6.27 (5.28) 5 (0–20)

^$^ Living with cancer (LWC), Family/Social issues (FSI), Children-related issues (CRI), Hereditary predisposition (HP), Emotion (E), Practical issues (PI); * *p*-value < 0.05, ** *p*-value < 0.01, *** *p*-value < 0.001, **** *p*-value < 0.0001.

**Table 3:** Multiple regression results of factors associated with the number of psychosocial needs.

Factors	Categories	Univariate Models (N = 392)			Final Multivariate Model (N = 380)		
		b	SE	*p* Value	b	SE	*p* Value
Intercept					4.53	1.08	<0.0001
Country	Germany (vs. France)	−1.29	0.5	0.01	-	-	
Age		−0.04	0.03	NS	−0.05	0.02	0.01
Education level	Secondary school or below	−0.39	0.51	NS	-	-	
Marital status	Others (than married/partnered)	0.16	0.54	NS	-	-	
Breast cancer diagnosis	Yes	−0.05	0.73	NS	1.05	0.60	0.08
HADS-Anxiety		0.79	0.05	<0.0001	0.78	0.05	<0.0001
HADS-Depression		0.6	0.07	<0.0001	-	-	
Loss of family member	No	0.43	0.58	NS	-	-	
Having children	Yes	−1.45	0.58	0.01	1.51	0.48	0.002
*R*^2^ (% of explained variance)					*0.40*		

NS: non-statistically significant; HADS: Hospital Anxiety and Depression Scale

**Table 4:** Literature review—Prevalence of needs.

Measure	% Prevalence	
PAHC		
% *Rated above threshold of* ≥*3*/*domain* ([40], Netherlands, various hereditary cancer syndromes)	Initial consultation	
Living with cancer (LWC)	84	
Hereditary predisposition (HP)	46	
Family/social issues (FSI)	45	
Children-related issues (CRI)	42	
Emotions (E)	29	
Practical issues (PI)	19	
GRACE *		
% *Rated at least quite a bit* ([38], UK, partly HBOC)	Initial consultation (Female)	1 month later/before test result disclosure (Female)
Implications of being at “increased” risk for cancer for other family members (FSI)	73	59
How one’s family would react if found to have an increased risk for cancer (FSI)	72	58
Will cope if found to have an increased risk of cancer (HP)	67	53
Possible future decisions about surgery(HP)	55	40
Impact of being at “increased” risk of cancer on lifestyle (PI)	50	44
Being able to get increased screening (HP)	49	27
Having to wait for information about one’s risk (HP)	47	39
Asking family members about family history (FSI)	41	35
Whether would be eligible for genetic testing (HP)	37	28
Completing the family history questionnaire (HP)	36	31
Not understanding the risk assessment process (HP)	24	19
MICRA		
% *Rated at least sometimes* ([39], Norway, ovarian cancer)	*BRCA1/2* carriers	Non-carrier affected/not and/or with/without family history
*Negative items*		
Worrying about my risk of getting cancer (again)	84	69
Uncertain about what the test result means for child(ren)/family’s cancer risk	84	43
Sad about my test result	69	5
Uncertain about what my test result means about my cancer risk	59	37
Anxious or nervous about my test result	50	8
Feeling a loss of control	50	8
Thinking that the test results have affected my work or family life	44	15
Upset about my test result	41	7
Frustrated that there are no definite cancer prevention guidelines for me	41	38
Guilty about my test result	31	2
Concerned about how my test results will affect my insurance status	31	10
Problems enjoying life because of my test result	25	6
Difficulty talking about my test results with family members	19	7
Difficulty making decisions about cancer screening or prevention (e.g., having preventive surgery or getting medical tests done)	18	14
Worrying that genetic counseling and testing process brought about conflict within my family	16	3
Regret about getting my test results	9	1
*Positive items*		
Happy about my test result	12	83
Relieved about my test result	16	82
Understanding clearly my choices for cancer prevention/early detection	84	68
Satisfied with family communication about my genetic test result	84	82
Feeling that family has been supportive during the genetic counseling and testing process	90	79
SUPPORT NEEDS *		
% *Rated high to very high* ([36], Australia, *BRCA1/2* tested)	≈1.7 years post test result	
Dealing with fears about developing cancer (LWC)	57	
Talking to other family members about having a faulty cancer protection gene (FSI)	54	
Obtaining information about the different options available to help manage your increased risk for cancer (HP)	51	
Dealing with uncertainty about the future (E)	49	
Talking to your children about their cancer risk (of women with children, *n* = 224) (CRI)	48	
Deciding how best to manage your increased cancer risk (HP)	43	
Talking with other women who have a faulty cancer protection gene (FSI)	41	
Finding someone who understands your situation (FSI)	39	
Dealing with feelings of sadness (E)	38	
Dealing with the impact that having a faulty gene has had on your family (FSI)	37	
Reassurance that the way you feel about your risk is normal (E)	35	
Dealing with the loss of family members who had breast cancer (FSI)	34	
Obtaining more information about the level of risk for breast cancer (HP)	32	
Understanding the information you have been given about your cancer risk (HP)	32	
Dealing with insurance issues that arise from having a faulty cancer protection gene (PI)	23	
Dealing with feelings of isolation (E)	22	
MICRA		
% *Rated at least rarely per domain* ** ([41], USA, *BRCA1/2* tested)	≈7 years post test result	
Distress	26	
Uncertainty	59	
Positive experience	51	

* Items classified according to Eijzenga et al. [17] needs’ categorization; ** Distress & Uncertainty: scores > 0; Positive experience: scores <20; PAHC: psychosocial aspects of hereditary cancer; GRACE: Genetic Risk Assessment Coping Evaluation; MICRA: Multidimensional Impact of Cancer Risk Assessment; HBOC: Hereditary Breast and Ovarian Cancer ; *BRCA1/2*genes.

**Table 5:** Literature review: correlates of concerns, difficulties, needs, care preferences, and unmet needs.

Study	Needs Correlates	
	Variables	Effect Sizes (Multivariate Results *)
Bennett [38] (N = 194)	Assessment timing	Level of GRACE concerns fell over time for “implications for family members if at increased risk” (Cohen’s h = 0.30); “how family would react if at increased risk” (Cohen’s h = 0.26); “possible decisions about surgery” (Cohen’s h = 0.28); “being able to get increased screening” (Cohen’s h = 0.38); and “having to wait to find out risk” (Cohen’s h = 0.16)
Bjornslett [39] (N = 354)	Age, education, having children, clinical, family history, test result, breast/ ovarian cancer, assessment timing, distress	Being a mutation carrier (β = 0.40) and higher traumatic anxiety (β = 0.48) related to the MICRA total score
Eijzenga [40] (N = 137)	Age, education level, marital status, having children, gender, clinical, index case, mutation in family, anxiety/ depression, previous contact with psychosocial worker	Having children associated with hereditary predispositions (OR = 2.56) and family/social issues (OR = 3.56); previous contact with psy associated to practical issues (OR = 0.38); anxiety/depression associated to higher all-domain needs, but partial correlations only significant for general emotions (r_p_ = 0.49), and family and social issues (r_p_ = 0.19)
Farrelly [36] (N = 279)	Age, education, marital status, having children, clinical, family history, assessment timing, someone to confide in	Age (β = −0.11), having a significant other to confide in (β = 0.14); time since notification of mutation status (β = −0.17) related to higher total need scores
Halbert [41] (N = 167)	Age, clinical, test result, timing	*BRCA1/2* carriers have more distress (OR = 3.96); shorter time since test disclosure related to more uncertainty (OR = 0.62)
Lumish [30] (N = 232)	Age, race, education, clinical, test result, assessment timing, genetics knowledge (only clinical and test results tested)	MICRA total scores were greater in the “no CA/mutation +” group. MICRA distress scores were greater in the “no CA/mutation +” group than in any of the other groups; the “CA/VUS” group had a higher MICRA distress scores than all of the other groups, other than the “no CA/mutation +” group. The “no CA/VUS” and the “CA/mutation +” groups had worse positive experience scores **
Oberguggenberger [37] (N = 137)	Breast/ovarian cancer diagnosis, test results	Significant difference in MICRA scales between positive versus negative results: distress: Cohen’s d = 0.57; uncertainty: Cohen’s d = 0.51; positive experiences: Cohen’s d = 0.96

* When available, only multivariate results were provided; ** Effect size could not be computed. GRACE: Genetic Risk Assessment Coping Evaluation; OR: odds ratio; VUS: variants of uncertain clinical significance.

## Supplementary Materials

The following are available online at http://www.mdpi.com/1660-4601/15/2/319/s1, Table S1: Literature search and article selection, Table S2: Study characteristics, Table S3: Measure characteristics as provided by the original developmental or psychometric study.
