# Supplementary material for: Patient-Centered Care in Breast Cancer Genetic Clinics
Source: Int J Environ Res Public Health. 2018 Feb 12;15(2):319. doi: 10.3390/ijerph15020319 (PMC5858388; doi:10.3390/ijerph15020319)
Supplement: Supplementary file 1 [file ijerph-15-00319-s001.pdf]

International Journal of
Environmental Research and Public Health

MDPI

Supplementary Materials

Table S1. Literature search and article selection.

**Literature Review on Period from 2000 to August 2017 (Last Check on 17/10/2017)**
Keywords:
((((((((genetic counseling) OR genetic testing) OR genetic predisposition)) AND ((breast neoplasm*) OR ovarian neoplam*)) AND ((((((((psychology(MeSH Subheading)) OR psychological adaptation(MeSH Terms)) OR need*(MeSH Terms)) OR unmet need*(MeSH Terms)) OR emotion*(MeSH Terms)) OR concern*(MeSH Terms)) OR quality of life(MeSH Terms) OR difficult*[MeSH Terms)) OR health service need*(MeSH Terms))) AND ("2000/01/01"(PDat) : "3000/12/31"(PDat)))) AND ((((questionnaire(MeSH Terms)) OR measure*(MeSH Terms)) OR scale(MeSH Terms)) AND ("2000/01/01"(PDat) : "3000/12/31"(PDat)))

Table S2. Study characteristics.

First Author	Year	Country	Objective	Study Design	N (% Response Rate) % Female	Age Mean (SD) (Range)	Period (Time Since Counselling/Testing)	Hereditary Syndromes; Healthy or Affected	Psychosocial Needs Measure	Other Measures
Bennett	2012	UK (Wales)	To assess detailed concerns and coping	Longitudinal prospective	194 (64% RR) 86% female	47.5 (13.5)	Before + 1 month after pre-counseling/before result disclosure	Partly HBOC/HNPCC % affected not provided	GRACE (Concerns)	GRACE (Coping)
Bjornslett	2015	Norway	To identify the women most vulnerable to experiencing psychological distress	Cross-sectional	354 (67% RR) 100% female	62.6 (11)	31 (18) months after testing	Affected with ovarian cancer (100%)	MICRA (Distress: $\alpha = 0.90$; Uncertainty: $\alpha = 0.81$; Positive experience: α=0.81 – confirmatory factor analyses adequate fit indices)	HADS, IES
Eijzenga	2015	Netherlands	To assess the prevalence and determinants of psychosocial problems	Cross-sectional	137 (52% RR) 82% female	47 (11)	Prior to genetic counselling (most)	Various hereditary syndrome; 71 (52%) affected	PAHC (Positive case if ≥1 item in a domain rated ≥3)	DT, HADS
Farrelly	2013	Australia	To identify variables that predict levels of unmet need	Cross-sectional	299 (45% RR) 100% female	46 (13.9)	1.7 (1.5) year after *BRCA1/2* positive test disclosure	Affected breast (37%), ovarian (6%), both (1.4%)	Modified version of Thewes et al. (2003) nine–item scale ($\alpha = 0.93$)	IES
Halbert	2011	USA	To assess the long-term reactions to *BRCA1/2* testing	Cross-sectional	167 (46% RR) 100% female	54.2 (9.8)	7.2 (2.2) years since test result	Affected breast cancer (50%)	MICRA (Distress: $\alpha = 0.87$; Uncertainty: $\alpha = 0.84$; Positive experience: $\alpha = 0.82$)	None
Lumish	2017	USA	To assess impact of panel gene testing in HBOC patients	Cross-sectional	232 (63% RR) 97% female	48.7 (12.8)	Months after testing: 13.3 (6.7) (affected); 12.5 (6.3) (Unaffected)	Affected breast or ovarian cancer (56%)	MICRA No psychometric information	IES, Satisfaction With Decision, Scale of Ambiguity Tolerance
Oberguggenberger	2016	Austria	To assess psychosocial outcomes and counselee satisfaction	Cross-sectional case-control study	137 (42% RR) 94% female	46.8 (12.7)	After counselling: 1.8 (0.9) year; 69% decided for testing	53% affected with cancer	MICRA (Scales $\alpha = 0.6–0.81$)	HADS, CWS, Satisfaction/Counselling/Decision, SF-12

MICRA: Multidimensional Impact of Cancer Risk Assessment; HBOC/HNPCC: Hereditary Breast and Ovarian Cancer/Hereditary Non-Polyposis Colon Cancer; GRACE: Genetic Risk Assessment Coping Evaluation; HADS: Hospital Anxiety and Depression Scale; CWS: Cancer Worry Scale; PAHC: Psychosocial Assessment in Hereditary Cancer; DT: explanation; IES: Impact of Event Scale.

Table S3. Measure characteristics as provided by the original developmental or psychometric study.

Measure	Aim-Targeted population	Number of items/scale-Domains	Development-Psychometrics	Scoring-Data analysis
Multidimensional Impact of Cancer Risk Assessment, MICRA Cella (2002)	To measure specific impact of result disclosure after genetic testing - Women tested for *BRCA1/2* mutation	25 items, incl. sections relevant when having children (two items) or having cancer (two items) - three scales: distress (six items), uncertainty (nine items), positive experience (four items) and two single items	IRT-based Rasch sequential residual factor analysis - scales (internal consistency): distress (α = .86), uncertainty (α =.77), and positive experiences (α =.75); differentiate between groups with different test result	Four-level Likert scale (never, rarely, sometimes, often) scored respectively 0, 1, 3 and 5; Sum of scores per scale and 21-item, raw or transformed (0–100) scores
Support needs questionnaire Farrelly (2013)	To assess level of unmet need - Women carriers of *BRCA1/2* mutation	16 items/one scale: information need about risk-reducing surgery and screening, communication with family members, social support, and psychological needs	Adapted from existing unmet needs questionnaires (Thewes, 2003); 16-item (internal consistency): α = 0.93	Five-point Likert scale (1 = no need, 2 = low need, 3 = moderate need, 4 = high need, 5 = very high need). Coded no need versus any need (low to very high); number of any need per woman
Genetic Risk Assessment Coping Evaluation, GRACE Phelps (2010)	To assess concerns and coping responses - between referral into clinical genetics service and notification of level of risk	11 items + open-ended 'other' (sources of stress) - degree of worry in response to each source and eight (and one other) coping strategies used to cope with each	Preliminary descriptive data. Relations between worries and coping strategies	4-point Likert scale - degree of worry (0=not at all/not applicable, 1 = a little, 2 = quite a bit, 3 = very much so). Dichotomous coding for coping strategies (absence/presence). Proportion of participants reporting level of worry about each potential source of stress and proportions of coping strategies used
Psychosocial Assessment in Hereditary Cancer, PAHC Eijzenga (2014)	To assess specific psychosocial problems related to cancer genetic counselling - Any hereditary cancer syndrome	26 items + open-ended 'other' - six domains: Hereditary predisposition (5); Practical issues (2); Family and social issues (6); General emotions (5); Living with cancer (5); and, for those who have children children-related issues (3) (only applicable to respondents having children) + 1 item on need for extra service by domain ("Would like to receive professional psychosocial support ?")	Development based on the European Organisation for Research and Treatment of Cancer Quality of Life Group guidelines. Screening properties of cut-off established against clinical interview	Four-point Likert scale - degree of difficulty (1 = not at all, 2 = a little, 3 = quite a bit, 4 = very much). Need for extra service (Yes/No). Prevalence by problem domain using cut-off ≥ 3 in at least 1 item by domain. Number of domains of needs

MICRA: Multidimensional Impact of Cancer Risk Assessment; GRACE: Genetic Risk Assessment Coping Evaluation; IRT: Item Response Theory; PAHC: Psychosocial Assessment in Hereditary Cancer; *BRCA1/2*: genes.
